# The Longitudinal Relationship Between Self-Esteem, Life Satisfaction, and Depressive and Anxiety Symptoms Among Chinese Adolescents: Within- and Between-Person Effects

**DOI:** 10.3390/bs15020182

**Published:** 2025-02-10

**Authors:** Zongqiao Han, Shuai Chen, Yan Zhou, Yanling Liu, Cheng Guo

**Affiliations:** 1 Research Center of Mental Health Education, Faculty of Psychology, Southwest University, Chongqing 400715, China; h112022306143741@email.swu.edu.cn (Z.H.); zh703123@email.swu.edu.cn (Y.Z.); ssq@swu.edu.cn (Y.L.); 2 Center for Studies of Psychological Application, School of Psychology, South China Normal University, Guangzhou 510631, China; chenshuai@m.scnu.edu.cn

**Keywords:** mental health, self-esteem, life satisfaction, cross-lagged panel model, random intercept cross-lagged panel model, developmental differences

## Abstract

Adolescents are especially vulnerable to experiencing depression and anxiety. This longitudinal study, from within- and between-person perspectives, explores how self-esteem relates to depressive and anxiety symptoms in Chinese adolescents and identifies the mediating factors impacting this relationship. Data were collected from 1025 junior and high school students in Southwestern China at three points over an 18-month period. This study utilized both traditional and random-intercept cross-lagged panel models to understand the dynamic developmental relationships. The general occurrence of depressive and anxiety symptoms increased longitudinally, with a more pronounced upward trend among female students. Between-person level analyses indicated bidirectional associations among self-esteem, life satisfaction, and depressive and anxiety symptoms. Moreover, life satisfaction emerged as a significant mediator. At the within-person level, self-esteem uniquely predicted both life satisfaction and subsequent depressive and anxiety symptoms. This study clarifies the longitudinal interplay between these constructs. Self-esteem, which denotes internal self-assessments, and life satisfaction, which denotes external evaluations of life, both significantly buffer the emergence of depressive and anxiety symptoms.

## 1. Introduction

Adolescence represents the critical developmental stage during which depressive and anxiety symptoms most frequently occur (Kessler et al., 2005; Merikangas et al., 2010), making adolescents a high-risk group for both conditions (WHO, 2024). Depression has emerged as a primary cause of disability and mortality among adolescents, while anxiety disorders typically develop during childhood or adolescence (WHO, 2023a, 2023b). Anxiety often coexists with depression. Adolescents with depression or anxiety have poorer social functioning (Copeland et al., 2021; Psychogiou et al., 2024), experience suicidal ideation (C. Kang et al., 2021), and exhibit lower prosocial tendencies (Yang et al., 2024) and life satisfaction (Essau et al., 2014). As competition intensifies, mental health issues among adolescents increase, with greater prevalence of depression and anxiety (Shorey et al., 2022). Consequently, it is essential to examine the factors and developmental processes associated with depressive and anxiety symptoms in adolescents.

The ongoing exploration of how self-esteem interacts with depressive and anxiety symptoms has been a major area of interest. During adolescence, the complex interplay between self-esteem and various social, cognitive, and emotional factors can significantly affect mental health (Costello & Maughan, 2015). These factors include not only the quality and quantity of peer and adult relationships but also broader ecological dimensions, such as school climate, sense of belonging, and perceived social support (de Arellano et al., 2023; Gaylord-Harden et al., 2007; B. X. Kang et al., 2024). Moreover, contemporary research underscores the importance of digital environments and online connectivity (Twenge et al., 2019), as hyper-connection can exacerbate stress, compare-based self-worth, and exposure to bullying or cyberbullying (Keles et al., 2020). Social pressures (e.g., social norms, social media use) related to culturally promoted aesthetic ideals contribute further to body dissatisfaction (Rajagopalan & Shejwal, 2014; Rodrigue et al., 2024). From the perspective of individuals’ self-perception, a longitudinal meta-analysis and an additional longitudinal study found that individuals possessing low self-esteem often experience increased depression and anxiety (Sowislo & Orth, 2013; Steiger et al., 2014). Although existing research has established a link between self-esteem and both depressive and anxiety symptoms, most studies have utilized cross-lagged panel models (CLPM) to investigate this relationship, yet only a few have examined and addressed the within-person and between-person effects involved. Meanwhile, adolescents’ self-esteem may influence their life satisfaction, while depressive and anxiety symptoms are often the outcomes of complex interactions between internal and external factors. Individuals’ evaluations of their lives are influenced by their objective life circumstances, indicating that life satisfaction plays a crucial mediating role in the association between self-esteem and depressive or anxiety symptoms. Accordingly, this study employs both the traditional CLPM and the random intercept cross-lagged panel model (RI-CLPM) across three waves of data to investigate the dynamic development of self-esteem and depressive or anxiety symptoms, including the mediating function of life satisfaction in this regard.

### 1.1. Self-Esteem, Depressive and Anxiety Symptoms

Self-esteem is an evaluative judgment based on self-perception, shaped by both cognitive and affective components, reflecting a person’s comprehensive evaluation and perception of their own worth (Leary & Baumeister, 2000). It is widely recognized as a fundamental indicator of psychological well-being (Rosenberg, 1965). Some researchers have distinguished between state self-esteem (Heatherton & Polivy, 1991) and trait self-esteem, with the former representing an individual’s subjective self-evaluation that fluctuates in response to emotional and contextual changes, while the latter reflects a relatively stable sense of self-worth. Existing research has consistently identified a significant negative relationship between self-esteem and depression (Bang et al., 2020). This relationship has been supported not only by cross-sectional studies (Nguyen et al., 2019) but also by longitudinal research (Gao et al., 2022). Given the frequent comorbidity between depressive and anxiety symptoms, scholars tend to discuss these conditions together. Self-esteem can impact both depressive and anxiety symptoms. According to Cognitive Theories of Depression (Beck, 1967), cognitive patterns, particularly self-perception, are critical contributors to depressive symptoms. Similarly, the Vulnerability Model (Ingram, 2003) posits that perceiving oneself as worthless predisposes individuals to depression. Derived from Terror Management Theory, the anxiety buffer hypothesis proposes that high self-esteem acts as a psychological defense against mental threats (e.g., negative life events or stress), protecting against anxiety and depressive symptoms (Solomon et al., 1991). Sowislo and Orth (2013) argued that low self-esteem heightens the risk of experiencing depression and anxiety, whereas high self-esteem supports a positive self-image and serves as a buffer against these emotional challenges. A longitudinal study using a traditional CLPM demonstrates that self-esteem uniquely predicts depression (Wouters et al., 2013). By contrast, the Scar Model advocates the reverse influence, suggesting that experiencing mental disorders can leave lasting “scars” on an individual’s personality, implying that self-esteem may adversely affected by these conditions (Lewinsohn et al., 1981).

Vulnerability and Scar Models should primarily be tested at the within-person level. This suggests that using traditional CLPMs may not be the most appropriate approach for exploring these relationships. Research employing RI-CLPMs to examine the association between self-esteem and depressive and anxiety symptoms has produced inconsistent findings concerning the direction of effects. At the within-person level, Jørgensen et al. (2023) found moderate vulnerability effects between the ages of 13 and 15 years, and small scar effects between the ages of 15 and 21 years. In exploring the above interplay within interpersonal relationships, Tran et al. (2023) found that depression predicts lower self-esteem, supporting the Scar Model. However, employing RI-CLPMs across three different data sets, Masselink et al. (2018) observed that self-esteem negatively influenced depressive symptoms but did not find evidence to support the scar effect. For anxiety symptoms, a longitudinal study employing an RI-CLPM revealed significant cross-lagged effects, in which self-esteem negatively predicts anxiety symptoms, but not vice versa (X. Q. Liu et al., 2022). However, another study reported bidirectional effects between self-esteem and anxiety symptoms (Li et al., 2023). In summary, there is substantial backing for the vulnerability model in most studies, but the Scar Model is only weakly supported (Orth & Robins, 2013).

Although previous studies employing RI-CLPM have yielded inconsistent results, these studies consistently indicate that low self-esteem is a significant risk factor for de- pression and anxiety. When considering contextual factors, some scholars have examined this relationship within specific interpersonal or sex contexts, while others have focused solely on the relationship between these variables. This difference in focus may have led to discrepancies in findings. From a methodological perspective, few studies have integrated both between- and within-person results to interpret the relationship among these constructs. This lack of integration may limit understanding of how these variables inter- act dynamically at different levels. Accordingly, this research aims to delineate the predictive relationship between self-esteem and both depressive and anxiety symptoms by formulating two main models—namely, one for depressive symptoms and one for anxiety—and examining the effects at both between- and within-person levels.

### 1.2. Life Satisfaction as Mediator

Life satisfaction reflects an individual’s subjective evaluation of their overall satisfaction with life (Diener et al., 2010). Various psychological theories acknowledge the interconnection between self-esteem and life satisfaction, each emphasizing distinct evaluative perspectives (Çivitci & Çivitci, 2009). Previous research and perspectives from positive psychology have identified self-esteem as an important indicator of life satisfaction (Moksnes & Espnes, 2013; Seligman, 2004; Szcześniak et al., 2022). Individuals with low self-esteem frequently experience self-doubt and perceive a reduced sense of control over their lives, which subsequently diminishes their life satisfaction. According to the sociometer theory (Leary & Baumeister, 2000), fluctuations in self-esteem reflect an individual’s perceived social value and acceptance in interpersonal interactions. When self-esteem increases, individuals experience greater acceptance and support in social interactions, promoting higher life satisfaction. Conversely, individuals with low self-esteem may exhibit heightened sensitivity to social evaluations and rejection (Minihan et al., 2023), resulting in decreased life satisfaction (Xing et al., 2024). However, longitudinal research has drawn inconsistent conclusions regarding the relationship between self-esteem and life satisfaction. For instance, a study involving Swiss adolescents demonstrated that self-esteem unidirectionally influences life satisfaction over time (Marcionetti & Rossier, 2019), whereas research on Korean adolescents identified a bidirectional cross-lagged effect between the two constructs (Kim & Nho, 2020).

Satici et al. (2016) proposed a mediating model in which life satisfaction serves as a cognitive mediator that reduces psychological vulnerability, including depressive and anxiety symptoms. Extensive research has associated life satisfaction with mental health outcomes, including depressive and anxiety symptoms. Individuals free from generalized anxiety symptoms report significantly higher life satisfaction than those experiencing such symptoms (Peixoto et al., 2023). Life satisfaction has also been shown to significantly negatively predict both depressive and anxiety symptoms (Mei et al., 2021). Cross-sectional studies have identified the impact of depressive and anxiety symptoms on life satisfaction (Duong, 2021; Foroughi et al., 2021), suggesting the possibility of a bidirectional path between these variables. For instance, one longitudinal study found that life satisfaction among older adults increases over time, while depression gradually diminishes (Lee et al., 2020). Another longitudinal study at the between-person level revealed cross-lagged effects between life satisfaction and both emotional disorders (Tang et al., 2024).

The relationships among the above constructs overlap with one another. According to Beck’s cognitive triad hypothesis for depression (Allen, 2003), depression originates from negative perceptions of oneself, the world, and future prospects. These three negative cognitions intertwine, driving depression. Low self-esteem causes individuals to feel worthless and perceive the world negatively, leading to diminished life satisfaction, difficulty finding fulfillment, and the development of hopelessness, depressive, and anxiety symptoms. Through a CLPM-based longitudinal analysis, it was determined that rumination mediates the relationship between self-esteem and depressive symptoms (Kuster et al., 2012). For adolescents, life typically encompasses domains such as school and family, and negative events in these areas can lower life satisfaction (Jovanovic, 2019). For instance, a longitudinal study from China found that bullying victimization mediates the role of self-esteem on depressive symptoms at the trait level (X. Wang et al., 2024). Furthermore, a longitudinal analysis of subjective well-being trajectories in college students found that self-esteem correlates with different patterns of subjective well-being, where stable and elevated levels of well-being are connected to lower depression risks (X. Q. Liu et al., 2024).

Although previous researchers have examined these relationships, the majority have employed cross-sectional methodologies, with few adopting a longitudinal approach. The reliance on single analytical models, such as CLPM or latent growth models, further limits the comprehensive understanding of these relationships, thereby creating significant gaps in the literature. While direct evidence supporting life satisfaction as a mediator is currently limited, ample longitudinal data suggest its potential role in linking self-esteem with depressive and anxiety symptoms. Consequently, this study is designed to bridge these gaps by employing (RI)-CLPMs to investigate both within- and between-person effects.

### 1.3. Current Study

Although earlier work has identified strong links among self-esteem, life satisfaction, and both depressive and anxiety symptoms, much of this evidence derives from cross-sectional designs or emphasizes between-person effects. This approach fails to capture dynamic within-person relationships over time, leaving the longitudinal relationships among these variables unclear. To address these limitations, the current study employs a longitudinal design with Chinese adolescents aged 13–18, encompassing distinct educational stages within China’s education system: junior middle school (ages 13–15) and senior high school (ages 16–18). Notably, in the first year of senior high school, students undergo subject specialization, a unique feature of the Chinese education framework that tracks students into different academic streams based on their strengths and career aspirations. The study utilizes both a traditional CLPM and RI-CLPM to investigate the between- and within-person dynamics linking self-esteem with depressive and anxiety symptoms over time. In addition, it examines whether life satisfaction mediates these associations. In doing so, this study tests the following hypotheses:

**H1:**  *At both the between- and within-person levels, there is a bidirectional relationship between self-esteem and depressive (H1a) and anxiety (H1b) symptoms.*

**H2:**  *At both the trait and state levels, there is a bidirectional relationship between self-esteem and life satisfaction.*

**H3:**  *Life satisfaction mediates the relationship between self-esteem and depressive (H3a) and anxiety (H3b) symptoms.*

## 2. Materials and Methods

### 2.1. Participants

This study utilized a longitudinal design over a period of 18 months, collecting data at three points at 6-month intervals. Participants were recruited from a secondary school that is representative of typical secondary education institutions in Sichuan Province, characterized by both middle and high school divisions, and a diverse student population. The selection was also based on the school’s accessibility and the existing collaborative relationship with its administration, which facilitated effective data collection. Taking into account the academic burdens faced by students and the logistical challenges of data collection, the students selected at Time 1 were from non-final year grades (9th grade and final-year high school). Ultimately, a total of 1025 students (34.8% male, 65.2% female; *M*_age_ = 15.33 years, *SD*_age_ = 1.48; 26.83% middle school, 73.17% high school) participated in this study’s school-organized longitudinal survey. At Time 1, participants were asked whether they were only children (10.7% only children, 89.3% non-only children). A total of 906 (33.7% male and 66.3% female) and 940 (33.9% male and 66.1% female) students participated at Time 2 and Time 3, respectively. Data were missing for 204 participants (19.9% of the total sample) across all three waves.

Ethical approval for this research was granted by the Institutional Review Board of the Psychology Department at Southwest University (IRB: H23175). Before the survey began, all participants provided informed consent. A trained graduate student collected the data in a standard classroom setting, with each participant completing the survey in about 20 min. Participants were briefed on the study’s objectives and reassured that their information would remain confidential and be used solely for research purposes.

### 2.2. Measures

#### 2.2.1. Self-Esteem

Self-esteem was measured using the Chinese version of the Self-Esteem Scale (Ji & Yu, 1999; Rosenberg, 1965). This 10-item instrument (e.g., “I feel that I have a number of good qualities”, “I take a positive attitude toward myself”) evaluates adolescents’ self-worth and acceptance. Each statement was evaluated on a 4-point Likert scale with anchors from 1 (“strongly disagree”) to 4 (“strongly agree”). Reverse scoring was utilized for Items 3, 5, 8, 9, and 10. Higher total scores correspond to greater self-esteem. Based on previous research (P. Wang et al., 1998), the scale has been suggested to present cultural differences, particularly regarding the expression of Item 8, which may not align well with the domestic context. To enhance the scale’s discriminative ability and psychometric properties, it is recommended that Item 8 be recoded as a positively scored item or deleted entirely. This study opted to remove Item 8. In this study, McDonald’s *ω* for the Self-Esteem Scale was 0.91 at T1, 0.92 at T2, and 0.94 at T3.

#### 2.2.2. Life Satisfaction

The Satisfaction With Life Scale (SWLS), developed by Diener et al. (2010), measures individuals’ life satisfaction levels and comprises 5 items (e.g., “I am satisfied with my life”, “The conditions of my life are excellent”). This scale demonstrates cross-cultural applicability and strong psychometric properties, making it a reliable tool for measuring life satisfaction. This study used the Chinese version of SWLS revised by K. T. Wang et al. (2009), which reflects robust reliability and validity. The self-report questionnaire employs a 7-point Likert scale ranging from 1 (strongly disagree) to 7 (strongly agree). The mean score across all items indicated individuals’ level of life satisfaction, with higher averages signifying greater satisfaction. In this sample, McDonald’s *ω* values were 0.92 at T1, 0.93 at T2, and 0.93 at T3.

#### 2.2.3. Depressive Symptoms

Depressive symptoms were evaluated using the Patient Health Questionnaire-9 (Kroenke et al., 2001), a 9-item self-report measure (e.g., “Little interest or pleasure in doing things”, “Feeling tired or having little energy”) that is aligned with the diagnostic criteria for major depressive disorders in the DSM-IV. Responses are recorded on a 4-point Likert scale from 0 (not at all) to 3 (nearly every day), with higher totals indicating more severe symptoms. Scores of 1–4 suggest minimal depression, 5–9 indicate mild depression, 10–14 represent moderate to moderately severe depression, and 20 or above signify severe depression. In this study, McDonald’s *ω* was 0.89 at T1, 0.92 at T2, and 0.94 at T3.

#### 2.2.4. Anxiety Symptoms

The Generalized Anxiety Disorder 7-item Scale (Spitzer et al., 2006), created by Spitzer and Kroenke, is a self-report measure evaluating the severity of generalized anxiety disorder, suitable for screening and as a diagnostic adjunct. Each of the seven items (e.g., “Feeling nervous, anxious, or on edge”, “Worrying too much about different things”) is rated from 0 (not at all) to 3 (nearly every day), producing a total score reflecting overall anxiety levels. Scores of 0–4 indicate no clinically significant anxiety, 5–9 suggest mild anxiety, 10–18 represent moderate anxiety, and 19 or above denote severe anxiety. In this sample, McDonald’s *ω* values were 0.95 at T1, 0.96 at T2, and 0.97 at T3.

##### Covariates

As sex and educational stage may influence the relationships among the variables in this study, they were included as control variables in the research model. During data collection, sex was coded as 1 for male and 2 for female. Additionally, participants’ educational stage was recorded, with middle school (Grades 1–3) coded as 1 and high school (Grades 4–6) coded as 2.

### 2.3. Data Analysis

#### 2.3.1. Missing Data

Using SPSS v29.0.1.0, Little’s MCAR (Little, 1988) test was conducted to examine the patterns of missing data. Results suggested that the missing variables followed MAR (*χ*^2^ = 70.75, *df* = 19, *p* < 0.001). A series of *t*-tests revealed that age (*t* = 8.64, *p* < 0.001), self-esteem (*t* = −1.98, *p* < 0.05), depressive symptoms (*t* = 3.89, *p* < 0.001), and anxiety symptoms (*t* = 3.18, *p* < 0.001) were MAR, whereas life satisfaction (*t* = −0.88, *p* = 0.378) was MCAR. Missing data were primarily owing to participant classes not completing the questionnaire or students transferring schools. Consequently, to address missing data in subsequent analyses, Mplus 8.3 applied the full information maximum likelihood approach.

#### 2.3.2. Descriptive Analysis

To advance understanding of adolescent mental health, it is essential to quantify the prevalence of depressive and anxiety symptoms within middle- and high-school cohorts. Accordingly, this study utilized SPSS v29 to conduct descriptive analyses of these symptoms, followed by Pearson correlation analyses to explore relationships among the key study variables.

#### 2.3.3. Measurement Invariance

As this study involved repeated measurements of participants across three time points, assessing measurement invariance was necessary. To this end, this study developed and analyzed configural, metric, and scalar invariance models. The nested longitudinal invariance models were assessed based on the recommendations of Chen (2007) that metric invariance is supported when changes in the Comparative Fit Index (ΔCFI ≤ 0.01), Root Mean Square Error of Approximation (ΔRMSEA ≤ 0.015), and Standardized Root Mean Square Residual (ΔSRMR ≤ 0.030) remain within these specified thresholds. For scalar and strict invariance, ΔCFI ≤ 0.01, ΔRMSEA ≤ 0.015, or ΔSRMR ≤ 0.010 are indicative of invariance across time (Stenling et al., 2018). As illustrated in Supplementary Table S1, the measurement model established scalar invariance, indicating that the observed changes over time were meaningful.

#### 2.3.4. Between-Person Effect (CLPM) and Within-Person Effect (RI-CLPM)

CLPM is frequently employed in longitudinal research because it assesses how variability between individuals in one construct influences subsequent changes in another. In doing so, it focuses on between-person effects without separating out within-person dynamics. Building on the CLPM, the RI-CLPM incorporates a random intercept that represents an individual’s initial status at the trait level, reflecting between-person differences, while the cross-lagged effects demonstrate how temporary deviations in one variable at a given time affect subsequent deviations in another variable for the same individual.

Utilizing Mplus 8.3, this study modeled two relational pathways—“self-esteem–life satisfaction–depressive/anxiety symptoms”—and investigated longitudinal associations at both trait and state levels using CLPM and RI-CLPM. This study used maximum likelihood estimation to analyze the models and then compared the two models to identify differences. A robust model requires evaluation using multiple indicators, including metrics such as CFI and Tucker–Lewis Index (TLI) above 0.90, and RMSEA and SRMR below 0.08. This study implemented a series of constraints in model construction (Orth et al., 2021). In the CLPM, the analysis began with an unconstrained model (CLPM1), followed by restrictions on autoregressive paths (CLPM2) and cross-lagged paths (CLPM3), ultimately applying constraints to both autoregressive and cross-lagged paths (CLPM4). For the RI-CLPM analysis, the process started with an unconstrained model (RI-CLPM1), followed by the gradual restriction of autoregressive paths (RI-CLPM2), addition of restrictions on cross-lagged paths (RI-CLPM3), and finally setting the within-person correlations to be equal (RI-CLPM4). ΔCFI ≤ 0.01 and ΔRMSEA ≤ 0.015 indicate no significant differences in fit between the compared models (Chen, 2008; Cheung & Rensvold, 2002). Finally, this study tested for mediation effects in the models using the Bootstrap method with the sample size set to 5000.

## 3. Results

### 3.1. Descriptive Analysis

As illustrated in Table 1, the proportion of middle school students displaying both depressive and anxiety symptoms increased over time, with female students demonstrating consistently higher prevalence rates than their male counterparts. At the high-school level, the number of female students experiencing depressive and anxiety symptoms exceeded that of their male peers at all three time points, with the lowest prevalence of both conditions observed at T2.

Table 2 displays the Pearson correlation coefficients for each time point. All variables were significantly correlated both within and across waves. In general, at every measurement occasion, higher levels of self-esteem and life satisfaction were associated with fewer depressive and anxiety symptoms. Moreover, self-esteem and life satisfaction exhibited a positive association, while depressive and anxiety symptoms were positively interrelated.

### 3.2. CLPM Results

Table 3 displays the fit indices for the CLPM and RI-CLPM, assessing the target variables while controlling for sex and educational stage. The fit indices for CLPM4 indicate a good fit, and there were no significant differences in model comparisons.

**Figure 1:**
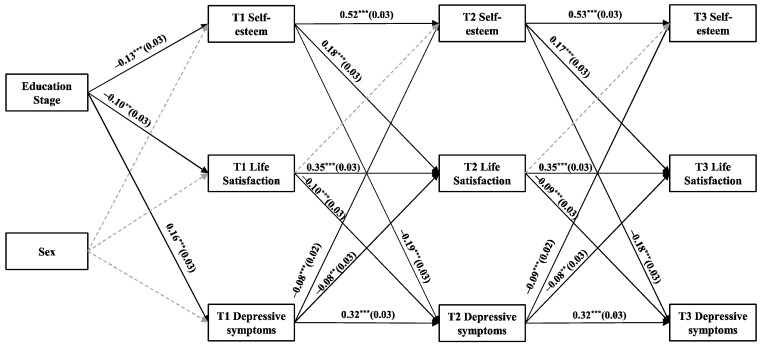
CLPM illustrating the relationships among self-esteem, life satisfaction, and depressive symptoms. All path coefficients are standardized, and gray dashed lines indicate non-significant pathways. Correlations within each time point and residuals are excluded for clarity. ** *p* < 0.01, and *** *p* < 0.001.

Figure 1 illustrates CLPM4 for depressive symptoms. T1 self-esteem positively predicts T2 life satisfaction (*β* = 0.18, *p* < 0.001), while T2 life satisfaction negatively predicts T3 depressive symptoms (*β* = −0.09, *p* < 0.001). Moreover, T2 life satisfaction significantly mediates the effect of T1 self-esteem on T3 depressive symptoms (*β* = −0.017, 95% CI [−0.027, −0.007]), thereby supporting H1a and H3a at the between-person level.

**Figure 2:**
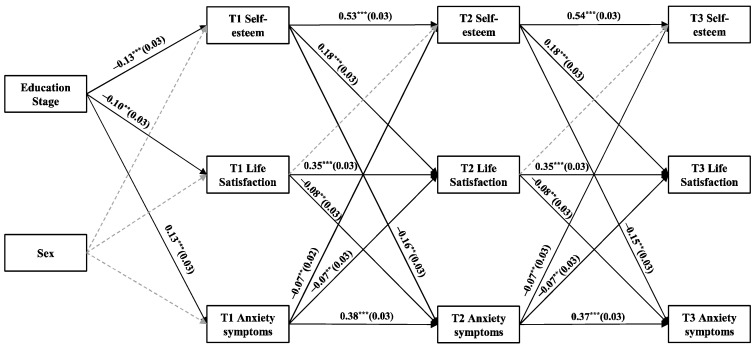
CLPM illustrating the relationships among self-esteem, life satisfaction, and anxiety symptoms. All path coefficients are standardized, and gray dashed lines indicate non-significant pathways. Correlations within each time point and residuals are excluded for clarity. ** *p* < 0.01, and *** *p* < 0.001.

Figure 2 presents CLPM4 for anxiety symptoms. T1 self-esteem positively predicts T2 life satisfaction (*β* = 0.18, *p* < 0.001), while T2 life satisfaction negatively predicts T3 anxiety symptoms (*β* = −0.08, *p* < 0.01). Additionally, the indirect effect of T1 self-esteem on T3 anxiety symptom through T2 life satisfaction is significant (*β* = −0.014, 95% CI = [−0.024, −0.004]), which supports H1b and H3b at the between-person level, but in both the depressive and anxiety symptoms models only a unidirectional effect path from self-esteem to life satisfaction was found, partially supporting H2.

This study also identified other mediating pathways, which are detailed in Supplementary Table S2. Overall, the results indicate that the positive pathway from self-esteem to life satisfaction negatively predicts both depressive and anxiety symptoms and exhibits a significant mediating effect. However, the reverse pathways do not demonstrate the same level of significance in either case.

### 3.3. RI-CLPM Results

This study used an RI-CLPM to conduct separate analyses of the relationship between self-esteem, life satisfaction, and depressive symptoms and that between self-esteem, life satisfaction, and anxiety symptoms. Sex and education stage were controlled for at the between-person level in the RI-CLPM. Table 3 presents the comparison indices for both models after applying constraints and reports the results for RI-CLPM4 for both the depressive and anxiety symptom models.

**Figure 3:**
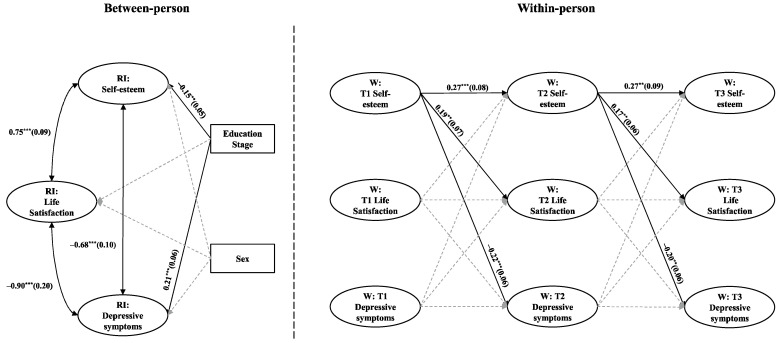
RI-CLPM illustrating the relationships among self-esteem, life satisfaction, and depressive symptoms. All path coefficients are standardized, and gray dashed lines indicate non-significant pathways. To enhance clarity, within-person level correlations are not displayed. ** *p* < 0.01, and *** *p* < 0.001.

**Figure 4:**
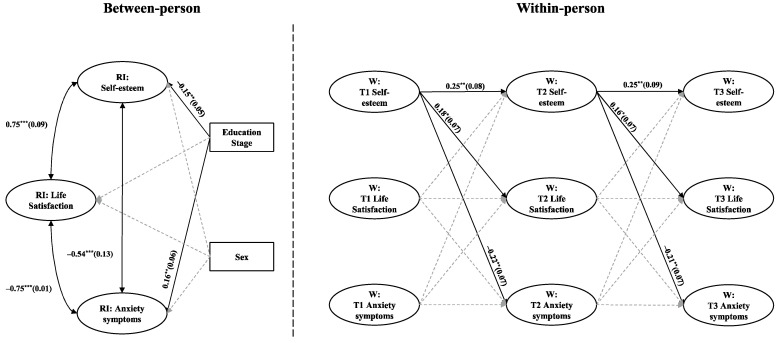
RI-CLPM illustrating the relationships among self-esteem, life satisfaction, and anxiety symptoms. All path coefficients are standardized, and gray dashed lines indicate non-significant pathways. To enhance clarity, within-person level correlations are not displayed. * *p* < 0.05, ** *p* < 0.01, and *** *p* < 0.001.

Figure 3 and Figure 4 illustrate the RI-CLPM4 results for depressive and anxiety symptoms, respectively, revealing similar patterns. At the between-person level, self-esteem is positively correlated with life satisfaction (r = 0.75, *p* < 0.001), while both self-esteem and life satisfaction are negatively correlated with depressive (r = −0.68 and −0.90, *p* < 0.001) and anxiety symptoms (r = −0.75 and −0.54, *p* < 0.001). At the within-person level, self-esteem at an earlier time point positively predicts life satisfaction later point (*β* = 0.19 and 0.17 for the depressive symptoms model and *β* = 0.18 and 0.16 for the anxiety symptoms model, *p* < 0.05) and negatively predicts both depressive (*β* = −0.22 and −0.20, *p* < 0.001) and anxiety symptoms (*β* = −0.22 and −0.21, *p* < 0.01). However, no significant within-person cross-lagged relationships were observed between life satisfaction and either depressive or anxiety symptoms. These results partially support H1a and H1b, as well as H2, at the within-person level, revealing only a unidirectional effect path from self-esteem to life satisfaction, depressive and anxiety symptoms, without finding a mediating role for life satisfaction.

## 4. Discussion

Although numerous studies have linked self-esteem, life satisfaction, and depressive and anxiety symptoms, only a limited number have explored their longitudinal dynamics. Addressing this gap, this study analyzed the prevalence of depressive and anxiety symptoms in adolescents, using advanced statistical models to explore the dynamic relationships between these variables. The findings can be summarized as follows. First, descriptive analysis showed that female students had higher prevalence rates of depression and anxiety than male students in both the middle- and high-school educational stages, with overall rates of both conditions increasing over time, especially for female students. Second, CLPM results revealed a bidirectional relationship between self-esteem, life satisfaction, and both emotional disorders, with life satisfaction strongly mediating this relationship. However, no reverse pathway from self-esteem to life satisfaction was identified. Third, RI-CLPM results showed significant correlations between self-esteem, life satisfaction, and both emotional disorders at the trait level. At the within-person level, self-esteem predicted life satisfaction and depressive and anxiety symptoms, but life satisfaction did not exert cross-lagged effects on these symptoms.

The results indicate that female students exhibit higher rates of depressive and anxiety symptoms compared to their male peers. Except for the anxiety detection rates in high school, the overall prevalence across the three time points shows a consistent upward trend, with female students experiencing a more rapid increase in depressive and anxiety symptoms than their male peers. Previous research on adolescent mental disorders has found that girls are particularly susceptible to socialization pressures and internalized emotions, which can significantly impact their mental health. When faced with emotional and social stressors, girls tend to exhibit different coping strategies than boys, often leading to greater emotional turmoil (Hyde et al., 2008). During adolescence, the pressures associated with gender role socialization intensify, exacerbating emotional distress among girls and increasing their vulnerability to emotional disorders (Nolenhoeksema & Girgus, 1994). Furthermore, in the Chinese context, the prevalence of depressive and anxiety symptoms among girls presents a more pronounced upward trend with age (X. Liu et al., 2024), suggesting that the challenges they face may become more complex and demanding as they mature.

The between-person results revealed a robust bidirectional relationship between self-esteem and both symptoms. Consistent with a recent study (Son et al., 2024), self-esteem and depressive symptoms had the strongest negative correlation during early adolescence at the one-year follow-up mark. A possible explanation is that individuals with higher self-esteem tend to experience fewer depressive and anxiety symptoms, as they are more likely to sustain positive self-evaluations and adopt adaptive attribution styles when encountering failure or challenges, reducing their vulnerability to depressive symptoms (Baumeister et al., 2003; Heimpel et al., 2002). Conversely, individuals with higher levels of depression or anxiety often experience declining self-esteem due to emotional distress and low confidence in the face of uncertainty (Brown & Marshall, 2013).

After controlling for trait-level effects, changes in self-esteem significantly and negatively predict subsequent changes in depressive and anxiety symptoms; this study provides empirical support for cognitive theories of depression, vulnerability theories, and the anxiety buffer hypothesis, which aligns with longitudinal research (Henriksen et al., 2017; Masselink et al., 2018). This study does not fully support the Scar Model, which suggests that depression negatively affects self-esteem. Notably, the CLPM indicated a bidirectional relationship between self-esteem and depressive and anxiety symptoms, whereas the RI-CLPM only identified a unidirectional path from self-esteem to both symptoms. This finding may be due to the limitations of the CLPM, which does not distinguish between trait and state effects. Self-esteem, depression, and anxiety are relatively trait-like constructs, potentially resulting in variations in the strength and direction of their relationship (Hamaker et al., 2015). A reasonable explanation is that, at trait level, self-esteem can form a long-term feedback loop with depressive and anxiety symptoms. However, at the state level, short-term boosts or drops in self-esteem trigger quick emotional responses before any reciprocal influence can register. In other words, while momentary increases or decreases in self-esteem may directly shape how one feels in the short run, the reverse pathway is overshadowed by broader, enduring vulnerabilities—such as stable cognitive styles or longstanding interpersonal stressors—that do not fluctuate as rapidly.

This study also found a bidirectional relationship between self-esteem and life satisfaction at the between-person level, aligning with previous research. Numerous studies have reliably demonstrated that higher self-esteem is associated with greater life satisfaction across diverse cultural backgrounds and age ranges (Diener & Diener, 1995). Similarly, people who experience greater life satisfaction tend to attribute positive experiences in their lives to their own efforts and value, thus enhancing their self-esteem (Kong et al., 2012).

Consistent with perspectives from positive psychology, this study found that, at the within-person level, changes in self-esteem positively predicted changes in life satisfaction, partially supporting H2. This aligns with previous findings suggesting that high self-esteem can prospectively predict success and satisfaction across various life domains (Orth & Robins, 2014). According to the sociometer theory, individuals whose self-esteem increases may receive positive feedback in social interactions, reinforcing their sense of self-worth and, in turn, enhancing life satisfaction. However, this study only found a unidirectional relationship at the within-person level, with no significant reverse prediction from life satisfaction to self-esteem. One possible explanation is that life satisfaction’s impact on self-esteem primarily occurs at the trait level, whereas self-esteem’s influence on life satisfaction operates at both the trait and state levels. Because life satisfaction reflects an individual’s overall evaluation of their life, and adolescence involves multiple spheres, such as school, peer relationships, and family life, contextual or situational factors may drive uncertain effects that, in turn, dilute any bidirectional relationship at the state level. Although self-esteem can fluctuate on a short-term basis and immediately shape how adolescents feel about their daily experiences, life satisfaction may require a longer timeframe to exert its influence, becoming evident only when considering more specific patterns of well-being.

The results also indicate a bidirectional association between life satisfaction and depressive and anxiety symptoms at the between-person level. This finding is consistent with previous research suggesting that individuals with lower life satisfaction are more likely to experience depression or anxiety and, conversely, that depression or anxiety can negatively impact life satisfaction (Martins et al., 2022). This may stem from individuals’ attribution styles (Diener & Chan, 2011), wherein they attribute unfortunate events in their lives to personal shortcomings and incompetence. Further, it could be related to individuals’ sense of efficacy (Hashemabady et al., 2022), wherein those with low levels of efficacy may lack confidence in their ability to cope with life events, leading to feelings of despair. Individuals with higher levels of depressive or anxiety symptoms tend to adopt negative cognitive processing styles (Lin et al., 2021), which may cause them to perceive their lives as unsatisfactory. However, at the state level, life satisfaction did not show any significant predictive pathways with depressive and anxiety symptoms, failing to support the notion of a dynamic relationship between life satisfaction and these emotional disorders. This suggests that the relationship between life satisfaction and depressive or anxiety symptoms may only exist at the trait level without a longitudinal connection over time.

The CLPM results demonstrated the expected effects, indicating that life satisfaction plays a strong mediating role in the longitudinal relationship between self-esteem and depressive and anxiety symptoms. This supports self-determination theory, which posits that emotional disorders are influenced by the fulfillment of three basic needs and complex mechanisms of well-being. Individuals with higher self-esteem may perceive more social support (Dündar & Yildiz, 2023), enhancing their life satisfaction. Furthermore, adolescents with diminished life satisfaction are at a higher risk of experiencing depression or anxiety (S. Wang et al., 2023), potentially due to their discontent and frustration with life. This may lead to the buildup of negative emotions, ultimately resulting in feelings of hopelessness and helplessness. In this study, when employing the RI-CLPM to differentiate between trait and state levels, the mediating role of life satisfaction did not remain significant.

### 4.1. Strengths, Limitations, and Future Directions

This study presents several key contributions to the field of adolescent mental health research within the Chinese context. First, it provides a comprehensive analysis of the prevalence rates of depressive and anxiety symptoms among Chinese adolescents, thereby offering an updated and culturally specific understanding of the current mental health landscape. Second, by employing a longitudinal design, the study investigates the dynamic and temporal relationships among self-esteem, life satisfaction, and depressive and anxiety symptoms, contributing to a more nuanced understanding of these interactions over time. Third, by advancing methodological rigor, this research distinguishes between within-person and between-person effects through the application of both CLPM and RI-CLPM. This dual-model approach elucidates the importance of separating trait-like and state-like variations when analyzing the interplay between psychological constructs. Furthermore, the study explores the mediating role of life satisfaction, identifying its significant influence on the relationship between self-esteem and depressive and anxiety symptoms over time. These findings underscore the critical role of life satisfaction as a mediator, highlighting its potential as a target for interventions aimed at enhancing psychological well-being. Collectively, these methodological and analytical advancements address previous inconsistencies in the literature and enhance our understanding of the complex interplay between self-esteem, life satisfaction, and emotional disorders during adolescence. Importantly, conducting this research within the Chinese cultural framework provides insights into factors such as collectivist family dynamics, high academic expectations, and societal stigma surrounding mental health, thereby informing the development of tailored mental health strategies and interventions that address these specific cultural and societal influences on adolescent well-being in China.

Notwithstanding these advantages, several limitations are as follows. Initially, although this study’s data analysis is feasible, the sample of adolescent participants is imbalanced in terms of educational stages and sex, which may limit the generalizability of the findings. Future research should aim for more balanced and diverse samples to prevent skewed outcomes and enhance representativeness. Second, from a methodological perspective, this study relied on self-report questionnaires for data collection. While questionnaires have historically proven to be robust and effective in psychology, the fast-paced nature of modern life may lead adolescents to respond less seriously, potentially compromising the authenticity of the data. Future research could employ innovative analytical approaches, such as analyzing adolescents’ daily-life (e.g., essays, diaries, or electronic device usage) using text analysis and machine learning to explore the relationships between self-esteem, depressive and anxiety symptoms, life satisfaction, and other potential variables. Third, as the data were collected from schools in a specific region of China, results may not generalize to other areas or cultures. Future investigations should gather data from more regions and cultural contexts to bolster external validity and build a more comprehensive understanding of adolescent well-being. Fourth, at the state level, this study identified only a unidirectional predictive relationship from self-esteem to life satisfaction, depressive and anxiety symptoms, implying that certain situational variables may have been overlooked. Future research should consider incorporating more context-specific factors into the analysis. Additionally, adopting more intensive data collection methods, such as experience sampling, could capture the nuanced day-to-day interplay between self-esteem, life satisfaction, and internalizing symptoms, thereby clarifying state-level effects. Finally, from a disciplinary standpoint, the variables, methods, and theories employed in this study remain predominantly within a psychological framework. However, adolescent life also requires insights from other academic fields to address mental health issues more comprehensively. Future research should thus consider additional interdisciplinary factors, integrating various elements not only at discrete time intervals but also through more intensive data collection. Such an approach would help identify important risk factors for adolescents within specific cultural contexts and illuminate the dynamic developmental changes and contextual differences underlying internalizing symptoms.

### 4.2. Implications

According to this study, the prevalence of depressive and anxiety symptoms is generally higher among female students than that among male students, with females exhibiting a more significant increase. This suggests that mental health interventions should place greater emphasis on the psychological well-being of female adolescents and develop targeted strategies to enhance their ability to cope with these challenges. Findings underscore the crucial role of self-esteem in relation to depressive and anxiety symptoms. Enhancing self-esteem can significantly improve mental health, especially as adolescents spend more time at school than at home. Therefore, school-level efforts are essential. Teachers can provide positive feedback to help students recognize their abilities and boost their confidence. Encouraging participation and self-expression in the classroom can aid in developing self-identity and enhancing self-esteem. Additionally, schools should create an inclusive and supportive environment wherein students feel safe and respected. The results also highlight the role of life satisfaction in the relationship between self-esteem and emotional disorders. Educators should focus on both academic performance and promoting social interaction by organizing activities that enhance social support. As some students may face familial challenges, fostering collaboration among families, schools, and communities is vital for ensuring students’ life satisfaction. Teachers can also incorporate mindfulness and gratitude exercises to cultivate positive emotions, thereby increasing life satisfaction and psychological resilience. Furthermore, a multi-tiered mental health support system should be established, integrating individual counseling, group therapy, and other support mechanisms to better assist students. At the state level, self-esteem and life satisfaction significantly predicted depressive and anxiety symptoms, while no reverse effects were observed. Interventions should therefore focus on enhancing state-level self-esteem and life satisfaction to more promptly prevent or alleviate depressive and anxiety symptoms. The absence of reverse effects also suggests that the influence of emotional states on self-esteem and life satisfaction may require a longer-term cumulative effect or may depend on the modulation of other contextual variables. Consequently, teachers should regularly monitor students’ changes and maintain close communication, while parents need to strike a reasonable work–family balance by devoting more time to their children and paying attention to the challenges and life events that adolescents face.

## 5. Conclusions

This study investigated the longitudinal dynamic relationships among self-esteem, life satisfaction, and depressive and anxiety symptoms. It expands existing research by distinguishing between the between- and within-person levels and interpreting the longitudinal relationships among these variables. The findings confirmed the existence of a bidirectional relationship between self-esteem, life satisfaction, and depressive and anxiety symptoms at the between-person level, with life satisfaction playing a significant mediating role in these longitudinal relationships. At the state level, fluctuations in self-esteem significantly impact changes in life satisfaction and depressive and anxiety symptoms, although no reciprocal pathways were observed. Additionally, when life satisfaction is considered at the within-person level, its mediating effect becomes non-significant. By providing fresh insights into the pathways linking self-esteem, life satisfaction, and mental health outcomes, this study advances the existing literature and aims to inspire further research in this domain.

## Figures and Tables

**Table 1:** Descriptive Analysis of the Prevalence of Depressive and Anxiety Symptoms.

	Depressive Symptoms				Anxiety Symptoms			
	Middle		High		Middle		High	
	Male	Female	Male	Female	Male	Female	Male	Female
T1	7 (2.55%)	27 (9.82%)	62 (8.27%)	104 (13.87%)	7 (2.55%)	15 (5.45%)	40 (5.33%)	77 (10.27%)
T2	12 (4.46%)	35 (13.01%)	49 (7.66%)	90 (14.06%)	9 (3.35%)	21 (7.81%)	31 (4.84%)	52 (8.13%)
T3	13 (4.74%)	43 (15.69%)	67 (10.06%)	99 (14.86%)	12 (4.38%)	30 (10.95%)	42 (6.31%)	63 (9.46%)

**Table 2:** Correlation Analysis of Main Variables.

	*M* ± *SD*	1	2	3	4	5	6	7	8	9	10	11	12
T1													
1. Self-esteem	2.89 ± 0.52	-											
2. Life satisfaction	4.69 ± 1.15	0.51 **	-										
3. Depressive symptoms	0.62 ± 0.59	−0.51 **	−0.34 **	-									
4. Anxiety symptoms	0.60 ± 0.68	−0.51 **	−0.36 **	0.83 **	-								
T2													
5. Self-esteem	2.87 ± 0.50	0.58 **	0.32 **	−0.36 **	−0.33 **	-							
6. Life satisfaction	4.56 ± 1.16	0.41 **	0.47 **	−0.32 **	−0.29 **	0.49 **	-						
7. Depressive symptoms	0.65 ± 0.60	−0.40 **	−0.33 **	0.43 **	0.45 **	−0.53 **	−0.33 **	-					
8. Anxiety symptoms	0.57 ± 0.67	−0.35 **	−0.28 **	0.41 **	0.44 **	−0.50 **	−0.29 **	0.85 **	-				
T3													
9. Self-esteem	2.84 ± 0.48	0.47 **	0.29 **	−0.32 **	−0.29 **	0.58 **	0.31 **	−0.36 **	−0.34 **	-			
10. Life satisfaction	4.70 ± 1.16	0.30 **	0.39 **	−0.25 **	−0.23 **	0.35 **	0.44 **	−0.24 **	−0.23 **	0.40 **	-		
11. Depressive symptoms	0.74 ± 0.63	−0.29 **	−0.25 **	0.37 **	0.35 **	−0.40 **	−0.24 **	0.44 **	0.41 **	−0.49 **	−0.35 **	-	
12. Anxiety symptoms	0.69 ± 0.73	−0.29 **	−0.25 **	0.36 **	0.39 **	−0.42 **	−0.26 **	0.45 **	0.48 **	−0.48 **	−0.34 **	0.86 **	-

Note: ** *p* < 0.01.

**Table 3:** Model Comparison Test of the CLPM and RI-CLPM.

Dependent Variable	*χ* ^2^	*df*	RMSEA	CFI	TLI	SRMR	ΔRMSEA	ΔCFI	ΔSRMR
Depressive symptoms									
CLPM1	111.324	21	0.065	0.969	0.921	0.033			
CLPM2	111.525	24	0.060	0.970	0.933	0.033	0.005	0.001	0.000
CLPM3	121.616	27	0.058	0.968	0.936	0.034	0.002	0.002	0.001
CLPM4	122.088	30	0.055	0.969	0.944	0.035	0.003	0.001	0.001
RI-CLPM1	25.657	6	0.057	0.993	0.940	0.017			
RI-CLPM2	25.657	9	0.042	0.994	0.966	0.017	0.015	0.001	0.000
RI-CLPM3	26.943	15	0.028	0.996	0.985	0.018	0.014	0.002	0.001
RI-CLPM4	54.573	21	0.039	0.989	0.971	0.023	0.011	0.007	0.005
Anxiety symptoms									
CLPM1	120.210	21	0.068	0.966	0.913	0.032			
CLPM2	121.016	24	0.063	0.967	0.926	0.033	0.005	0.001	0.001
CLPM3	127.910	27	0.060	0.966	0.931	0.034	0.003	0.001	0.001
CLPM4	133.027	30	0.058	0.965	0.937	0.036	0.002	0.001	0.002
RI-CLPM1	25.333	6	0.056	0.993	0.941	0.017			
RI-CLPM2	25.333	9	0.042	0.994	0.967	0.017	0.014	0.001	0.000
RI-CLPM3	27.751	15	0.029	0.996	0.984	0.018	0.013	0.002	0.001
RI-CLPM4	51.300	21	0.038	0.990	0.973	0.025	0.009	0.006	0.007

## Data Availability

The datasets generated and analyzed during the current study are not publicly available but are available from the corresponding author upon reasonable request.

## Supplementary Materials

The following supporting information can be downloaded at: https://www.mdpi.com/article/10.3390/bs15020182/s1, Table S1: Fit statistics for measurement model and tests of measurement invariance; Table S2: Indirect effect in CLPM.
