# Supplementary material for: The Longitudinal Relationship Between Self-Esteem, Life Satisfaction, and Depressive and Anxiety Symptoms Among Chinese Adolescents: Within- and Between-Person Effects
Source: Behav Sci (Basel). 2025 Feb 10;15(2):182. doi: 10.3390/bs15020182 (PMC11851692; doi:10.3390/bs15020182)
Supplement: Supplementary file 1 [file behavsci-15-00182-s001.zip › behavsci-3421734-supplementary.pdf]

The Longitudinal Relationship between Self-Esteem, Life Satisfaction, and Depressive and Anxiety Symptoms among Chinese Adolescents: Within- and Between-Person Effects

Table S1 Fit statistics for measurement model and tests of measurement invariance
Table S2 Indirect effect in CLPM

Table S1

Supplementary Table S1. Fit statistics for measurement model and tests of measurement invariance

	χ2	*df*	RMSEA	CFI	TLI	SRMR	ΔRMSEA	ΔCFI	ΔSRMR
Self-Esteem									
CONFIG	663.743	270	0.038	0.977	0.970	0.098			
METRIC	746.686	286	0.040	0.973	0.967	0.104	0.002	0.004	0.006
SCALAR	878.385	302	0.043	0.966	0.961	0.107	0.003	0.007	0.003
Life satisfaction									
CONFIG	255.441	72	0.050	0.980	0.970	0.028			
METRIC	270.829	80	0.048	0.979	0.972	0.035	0.002	0.001	0.007
SCALAR	289.295	84	0.049	0.977	0.972	0.034	0.001	0.002	0.001
Depressive symptoms									
CONFIG	1124.387	294	0.052	0.944	0.933	0.033			
METRIC	1183.085	310	0.052	0.941	0.933	0.038	0.000	0.003	0.005
SCALAR	1252.674	326	0.053	0.937	0.932	0.039	0.001	0.004	0.001
Anxiety symptoms									
CONFIG	968.201	165	0.069	0.957	0.945	0.026			
METRIC	994.737	177	0.067	0.956	0.948	0.029	0.002	0.001	0.003
SCALAR	1070.329	189	0.067	0.953	0.948	0.030	0.000	0.003	0.001

Table S2

Supplementary Table S2. Indirect effect in CLPM

Indirect path	beta	standardized beta	95%CI
T1SE-T2DEP-T3LS	0.034	0.015	[0.005, 0.025]
T1SE-T2LS-T3DEP	-0.02	-0.017	[-0.027, -0.007]
T1DEP-T2LS-T3SE	-0.003	-0.003	[-0.007, 0.001]
T1DEP-T2SE-T3LS	-0.028	-0.014	[-0.023, -0.006]
T1LS-T2DEP-T3SE	0.004	0.009	[0.002, 0.015]
T1SE-T2ANX-T3LS	0.023	0.01	[0.002, 0.018]
T1SE-T2LS-T3ANX	-0.019	-0.014	[-0.024, -0.004]
T1ANX-T2LS-T3SE	-0.002	-0.003	[-0.006, 0.001]
T1ANX-T2SE-T3LS	-0.02	-0.012	[-0.020, -0.003]
T1LS-T2ANX-T3SE	0.002	0.006	[0.001, 0.0011]
